# A Functional Mutation in *KIAA1462* Promoter Decreases Glucocorticoid Receptor Affinity and Affects Egg-Laying Performance in Yangzhou Geese

**DOI:** 10.3390/ijms19051531

**Published:** 2018-05-21

**Authors:** Mengyuan Xia, Wei Wei, Zaohang Jiang, Dandan He, Zhen Li, Shigang Yu, Qiushi Wang, Honglin Liu, Jie Chen

**Affiliations:** College of Animal Science and Technology, Nanjing Agricultural University, Nanjing 210095, China; 2014105074@njau.edu.cn (M.X.); wei-wei-4213@njau.edu.cn (W.W.); 2014105002@njau.edu.cn (Z.J.); 2014105001@njau.edu.cn (D.H.); 2014105029@njau.edu.cn (Z.L.); 2012205002@njau.edu.cn (S.Y.); 2015105001@njau.edu.cn (Q.W.); liuhonglin@njau.edu.cn (H.L.)

**Keywords:** *KIAA1462*, SNP, promoter, Yangzhou goose, laying performance

## Abstract

The identification of genetic markers is valuable for improving the egg-laying performance in goose production. The single-nucleotide polymorphism (SNP) rs1714766362 in an intron of the goose *KIAA1462* gene was found to be relevant to laying performance in our previous study. However, its function remains unclear. In this study, the full-length coding sequence of *KIAA1462* gene was firstly characterized in Yangzhou geese. Q-PCR (Quantitative Real Time Polymerase Chain Reaction) results showed that *KIAA1462* was highly expressed in the liver, ovary, and mature F1 follicles. For SNP rs1714766362, geese with the AA genotype showed better laying performance than the TT ones and exhibited a higher *KIAA1462* expression level in the ovary. Gain- and loss-of function experiments in granulosa cells revealed that *KIAA1462* affected the expression of the apoptosis marker gene caspase-3. Considering that rs1714766362 locates in an intron area, we compared the *KIAA1462* promoter regions of AA and TT individuals and identified the SNP c.-413C>G (Genbank ss2137504176), which was completely linked to SNP rs1714766362. According to the transcription factor prediction results, the glucocorticoid receptor (GR) would bind to the SNP site containing the C but not the G allele. In this study, we proved this hypothesis by an electrophoretic mobility shift assay (EMSA). In summary, we identified a novel mutation in the promoter of *KIAA1462* gene which can modulate GR binding affinity and affect the laying performance of geese.

## 1. Introduction

Goose possesses many properties, including rapid growth, disease resistance, high liver lipid storage capacity, and are easily fed with coarse fodder. The Yangzhou goose is one of Chinese indigenous goose breeds, mainly distributed in Jiangsu province of China. In recent years, it has attracted increasing attention because of its high yield of breast meat and low caloric content. However, the goose industry is largely hindered by goose poor laying performance, which is characterized by seasonal egg laying, strong incubation tendency, and low egg-laying rate. It takes approximately 18 days for large white follicles to develop into mature F1 follicle [1], and, on average, oviposition intervals last 46.8 h (36–55 h). Avian egg laying is coordinated by hormones secreted by the pituitary gland and ovarian follicles [2] and by receptors on the surface of the follicular cells [1]. Follicle development involves several processes, including proliferation, differentiation, and apoptosis of follicular cells. Studies have provided insights into the endocrine regulatory mechanisms of follicular maturation and ovulation and have identified a large number of hormones which perform various functions in goose egg laying.

In our previous study, a laying-related SNP rs1714766362 was found in an intron of the goose *KIAA1462* gene. *KIAA1462* product was also designated JCAD (Junctional protein associated with coronary artery disease), which is a novel molecular component of VE-cadherin-based endothelial cell–cell junctions [3], and was identified by restriction site-associated DNA (RAD) sequencing [4]. However, the molecular mechanism of the SNP effect on egg laying remains unexplained. *KIAA1462* gene does not have obvious domain structures with a predictable function. In vitro and in vivo studies suggest that *KIAA1462* plays an essential role in pathological angiogenesis, and the SNPs in the human *KIAA1462* gene have been shown to be associated with coronary artery disease [5]. A genetic variant in *KIAA1462* gene was also found to play a role in meiosis, therefore leading to a better understanding of pregnancy loss [6]. Besides, *KIAA1462* gene is mutated in ovarian cancer and may play a pathogenic role in ovarian serous borderline tumors [7]. These observations highlight the potential role of *KIAA1462* in human reproduction. Nevertheless, there is no report about *KIAA1462* in any pathways influencing goose laying performance.

Laying performance is affected by various factors, such as food, condition, and hormones. Corticosterone, and in particular glucocorticoids, are one kind of hormones influencing birds laying performance by affecting appetite. Several studies previously performed in chicks can help understand the process of egg laying in geese. For instance, some studies showed that hens fed corticosterone or infused with corticosterone showed ovary regression and reduced weight gain and egg production [8,9]. Glucocorticoids downregulated the expression of appetite-related genes in the hypothalamus of HFD (High-Fat Diet) -fed chicks by an AMPK (Adenosine 5‘-Monophosphate (AMP)-Activated Protein Kinase) –neuropeptide Y signaling pathway and a TOR (Target of Rapamycin) pathway [10,11].

On the basis of our previous RAD (Restriction-Site Associated DNA) sequencing results, that identified a laying-related SNP rs1714766362 in an intron of the goose *KIAA1462* gene, and the role of *KIAA1462* gene mutations in reproduction, we raised the hypothesis that this SNP might influence goose laying performance. The present study aims to explain the functional mechanism of this mutation using Yangzhou geese. To do this, the cDNA sequence of *KIAA1462* gene was firstly cloned, the gene expression profiles were analyzed in different tissues, and the differential expression of *KIAA1462* gene in different genotypes of SNP rs1714766362 in Yangzhou geese’s ovarian was detected. The role of *KIAA1462* gene in regulating granulosa cell apoptosis was investigated by gain- and loss-of-function experiments. We further identified a novel rs1714766362-linked mutation, namely, c.-413C>G, functionally altering glucocorticoid receptor (GR) binding affinity and transcriptional activity. We hope that this study will help identify genes or molecular markers effective for the improvement of the laying performance of geese.

## 2. Results

### 2.1. SNP (Single Nucleotide Polymorphisms) Genotyping and Association Analysis

By allele-specific PCR (AS-PCR), the rs1714766362 SNP was genotyped in a total of 256 geese. Three different genotypes, namely, TT, TA, and AA, were identified (Figure S1). The frequency of the A allele (0.81) was significantly higher than that of the T allele (0.19). The frequency of the AA genotype individuals was higher than that of the TA and TT genotype individuals (0.66 compared with 0.29 and 0.05, respectively). The association analysis between different genotype individuals and the level of egg production was analyzed by one-way analysis of variance, using SPSS 16.0 software (SPSS, Inc., Chicago, IL, USA). The results showed that geese with the AA genotype laid a significantly larger number of eggs (79.60 ± 11.08) than those with the TT genotype (73.00 ± 9.61) (*p* < 0.05). Furthermore, the eggs number of individuals with the AA genotype was larger than that of individuals with the TA (77.61 ± 11.11) genotype, although the difference was not significant (Table 1). This indicates that the rs1714766362 SNP of *KIAA1462* is related to the laying performance in Yangzhou geese.

### 2.2. Sequence Characterization and Phylogenetic Relationships among Species of Yangzhou Geese KIAA1462 Gene

The full-length coding sequence of goose *KIAA1462* (4029 bp length including the stop codon TGA) was assembled from three PCR-amplified overlapped cDNA fragments (1305, 1498, and 1664 bp, respectively) using the Seqman program of the DNASTAR software (DNASTAR Inc., Madison, WI, USA). According to the prediction result, this cDNA encodes a protein of 1342 amino acids with a theoretical molecular mass of 147.0519 KDa, and its isoelectric point is 8.62. The homology analysis of goose *KIAA1462* gene coding sequence (CDS) compared with the *KIAA1462* gene of other species is shown in Table 2. To provide convenient and intuitive results, we used the amino acid sequences of the species mentioned in the table to construct a phylogenetic tree by the neighbor-joining method (Figure S2). The phylogenetic tree suggests that the species could be clustered into two groups. We found that *KIAA1462* protein is conserved among birds and that the *KIAA1462* protein of Yangzhou geese is most closely related to the duck corresponding protein among the bird species examined (Figure S2).

### 2.3. KIAA1462 mRNA Expression Profile in Yangzhou Geese Tissues

**Figure 1:**
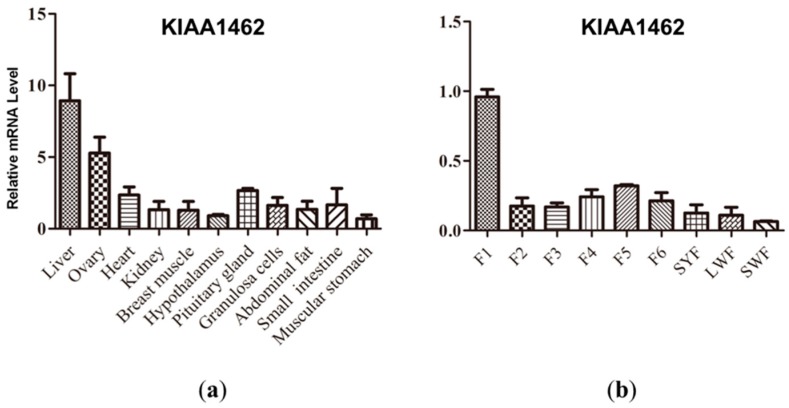
*KIAA1462* mRNA expression profile in Yangzhou geese tissues. (**a**) qPCR was performed to evaluate the expression levels of *KIAA1462* mRNA in 11 tissues (**b**). SYF: small yellow follicle; LWF: large white follicle; SWF: small white follicle. F1–6: follicle developmental stages.

The expression levels of *KIAA1462* mRNA in 11 tissues (kidney, ovary, small intestine, liver, abdominal fat, muscular stomach, breast muscle, heart, hypothalamus, pituitary gland, and granulosa cells) were evaluated by qPCR. The results showed that *KIAA1462* was ubiquitously expressed in the 11 tested tissues (Figure 1a). The higher expression levels were detected in the liver and ovary compared to the other tissues (Figure 1a). In addition, the level of *KIAA1462* mRNA was determined in granulosa cells isolated from developing follicles. *KIAA1462* gene was expressed in all developing stages and showed the lowest mRNA level in the small white follicles. The mRNA expression level varied slightly during the maturation process from large white follicle to F2 follicle, with a transient increase in F5 follicle. The highest level of *KIAA1462* mRNA was present in mature F1 follicle (Figure 1b).

### 2.4. KIAA1462 mRNA Level Differs in Individuals with Different Genotypes

**Figure 2:**
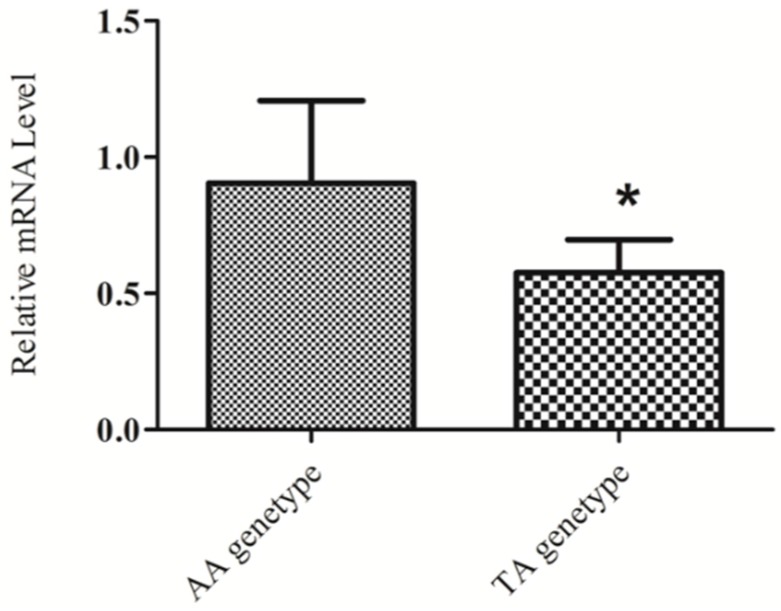
The mRNA expression levels of *KIAA1462* gene in the ovary of AA and TA genotype geese. Two-tailed student’s *t*-tests were performed to compare gene expression in the two genotypes. The error bars represent the standard error of the mean. * *p* < 0.05.

The expression level of *KIAA1462* in the ovary was compared in individuals presenting the two genotypes (TA and AA) for SNP rs1714766362. The relative expression results indicated that geese with the AA genotype had a higher mRNA expression level of *KIAA1462* in the ovary than the TA genotype individuals (*p* < 0.05) (Figure 2). The TT genotype individuals were difficult to identified, so we could not obtain a sufficient amount of ovary tissue to analyze the *KIAA1462* expression level. In our previous study, the AA genotype individuals showed better egg-laying performance than the TA and TT individuals [4]. In this paper, a significant difference in egg production was also observed between AA and TT individuals (Table 1). The best egg-laying performance and highest mRNA levels observed in AA genotype individuals indicated that the mRNA expression level of *KIAA1462* was positive correlated with egg-laying performance in Yangzhou geese.

### 2.5. The mRNA Level of Caspase-3 Is Negative Regulated by KIAA1462

In order to estimate the effect of *KIAA1462* on egg laying, we employed gain- and loss-of-function experiments to study *KIAA1462* influence on the apoptosis of follicle granulosa cells. Caspase-3 activation is critical for apoptosis [12]; therefore, it was reasonable to determine the expression of apoptosis-related caspase-3 in the target tissues to study the effects of certain hormones or supplements affecting egg laying [13,14]. The caspase-3 mRNA level determined by qPCR was thus used to evaluate the effect of the varying *KIAA1462* expression on apoptosis.

**Figure 3:**
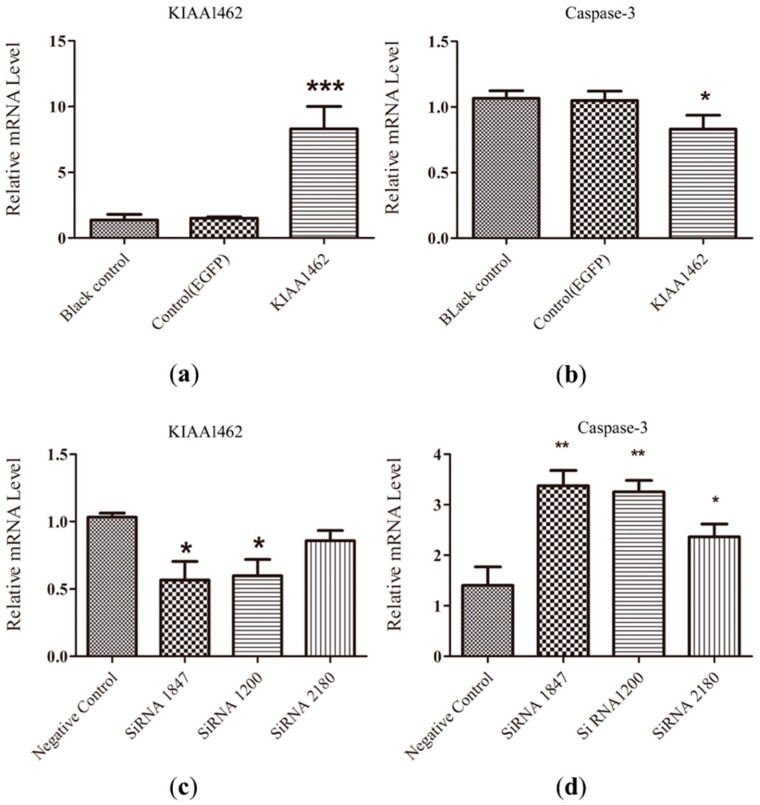
The mRNA level of caspase-3 is affected by *KIAA1462* in granulosa cells of Yangzhou goose. (**a**) *KIAA1462* mRNA levels were quantified by qPCR 24 h after transfection with the pEGFP-*KIAA1462* expression construct, EGFP plasmid (Control (EGFP)), and in untransfected cells (Blank control), respectively. (**b**) caspase-3 mRNA levels were quantified 24 h after transfection with the expression construct pEGFP-*KIAA1462*, EGFP plasmid (Control (EGFP)), and in untransfected cells. (**c**) *KIAA1462* mRNA levels were quantified 24 h after transfection with siRNA1847, siRNA1200, siRNA2180, control siRNA, and in untransfected cells (Blank control). (**d**) caspase-3 mRNA levels were quantified 24 h after transfection with ssiRNA1847, siRNA1200, siRNA2180, control siRNA, and in untransfected cells; * denotes significant differences (*p* < 0.05), ** denotes highly significantly differences (*p* < 0.01), *** denotes *p* < 0.001, two-tailed student’s *t*-test.

For the gain-of-function experiment, a vector inducing the overexpression of *KIAA1462* was transfected into the granulosa cells of follicles. Twenty-four hours later, *KIAA1462* mRNA level was distinguishably higher in the transfected cells than in the two control groups (Figure 3a), while caspase-3 mRNA levels were significantly decreased compared with the control groups (Figure 3b). For the loss-of-function experiment, knockdown of *KIAA1462* was achieved by using three sets of siRNAs. The results showed that siRNA1200 and siRNA1847 could significantly reduce the amount of *KIAA1462* mRNA (Figure 3c). Compared with the control groups, caspase-3 mRNA level was significantly increased in the *KIAA1462* knockdown groups treated with siRNA1847, siRNA1200 (*p* < 0.01), and siRNA2180 (*p* < 0.05) (Figure 3d).

### 2.6. Direct Sequencing of the 5′ Flanking Region of KIAA1462

The above results revealed that SNP rs1714766362 was correlated to differential *KIAA1462* expression levels and laying performance. According to a location analysis, SNP rs1714766362 located in intron 2 of the *KIAA1462* gene, which does not play a role in translation and contributes to the structure of the encoded protein. It is possible that functional mutation functions in linkage disequilibrium. Mutations in introns may affect splice or branch sites or may linkage with other mutations which situated in regulatory regions of a gene that affect the amount of produced transcripts or translation. Therefore, we tried to screen the regulatory region of *KIAA1462* gene in the attempt to find a linked functional mutation and explain the mRNA changes observed in the different genotypes of SNP rs1714766362. We sequenced and compared the 3 kb promoter region of *KIAA1462* in individuals with the AA genotype and the TT genotype for SNP rs1714766362 based on the goose genomic DNA sequence and the goose mRNA sequence (NCBI accession nos. NW_013185782.1, XM_013191711.1). The result suggested that there was a novel mutation, namely, c.-413C>G, located in the regulatory region 413 bp upstream of the start codon, which was completely linked with SNP rs1714766362 in the *KIAA1462* gene. The SNP c.-413C>G was deposited in Genbank dbSNP (ss2137504176). The c.-413 C allele corresponds to the A allele of SNP rs1714766362, and the c.-413 G links corresponds to the T allele (Figure S3). This complete linkage was further verified in 100 individuals.

### 2.7. The c.-413C>G Mutation Causes Allele-Specific Binding of GR

**Figure 4:**
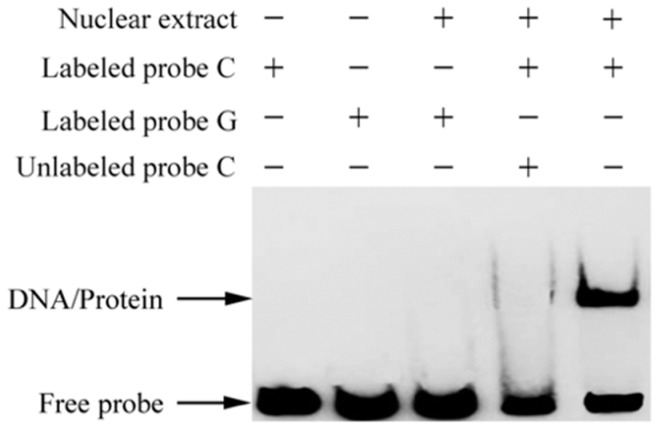
SNP (c.-413C>G) affects the binding affinity of nuclear proteins. Electrophoretic mobility shift assay (EMSA) was performed using a 31 bp labeled probe or unlabeled probe of the SNP (c.-413C>G) alleles. The unlabeled probe C was used as a competitor. The black arrow indicates the shifted band specific to the C allele of SNP (c.-413C>G).

To investigate whether this novel mutation modulates the binding affinity of the flanking sequence to transcription factors, we conducted an electrophoretic mobility shift assay (EMSA) using nuclear extracts of Yangzhou goose granulosa cells. The results demonstrated that the sequence containing the C allele exhibited stronger binding affinity to a nuclear protein(s) than the G allele (Figure 4).

**Figure 5:**
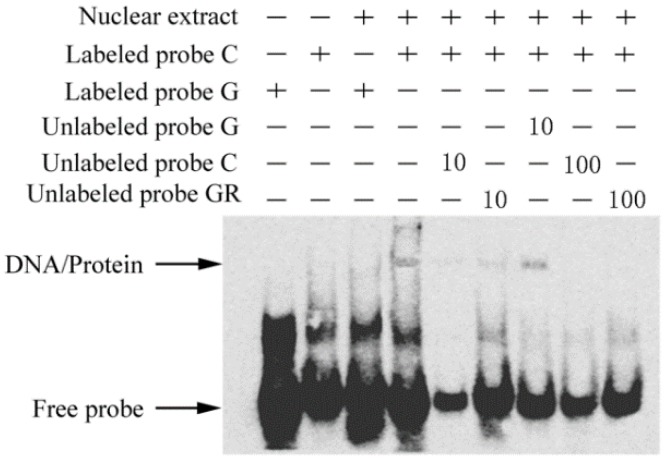
Binding of the transcription factor glucocorticoid receptor (GR) to the C allele of SNP (c.-413C>G). EMSA was performed using the labeled C or G alleles of SNP (c.-413C>G) and nuclear extract from Yangzhou geese granulosa cells. Unlabeled consensus oligonucleotides of C or G and transcription factor GR were used as competitors. DNA/protein indicates the shifted band.

The transcription factor prediction tool MatInspector (http://www.genomatix.de/products/MatInspector/index.html) [15] was then used to search for the potential transcription factors binding to the target binding site. A putative binding affinity of the flanking sequence to glucocorticoid receptor (GR) was predicted for the C allele but not for the G allele. We further performed a competitor assay of EMSA using non-labeled GR-consensus oligonucleotides. It was confirmed that GR-consensus oligonucleotides could effectively inhibit the binding of nuclear protein(s) to the labeled C allele (Figure 5). The results suggested that the C allele allows GR binding, while the G allele prevents this binding.

### 2.8. GR mRNA Expression Profile in Yangzhou Geese Tissues

**Figure 6:**
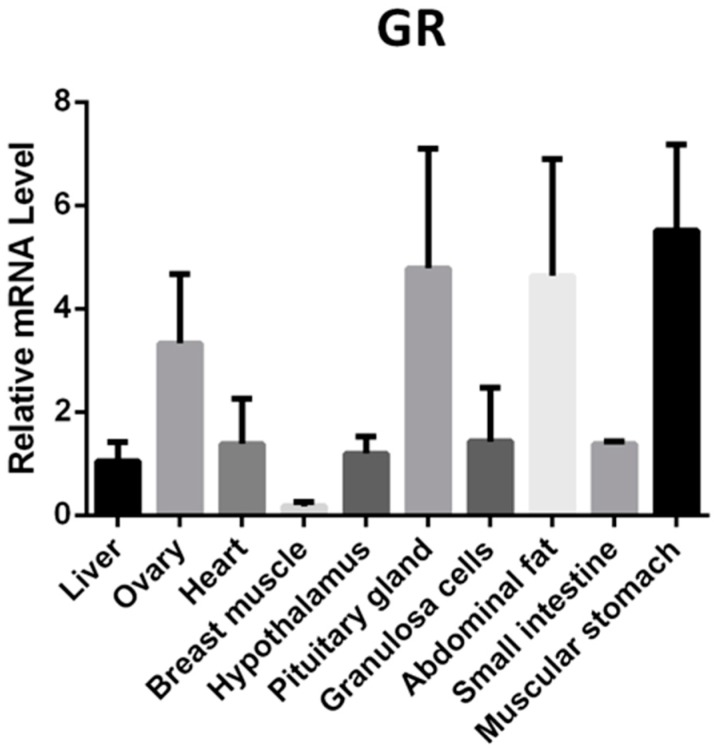
GR mRNA expression profile in Yangzhou geese tissues. qPCR was performed to evaluate the expression levels of GR mRNA in liver, ovary, heart, breast muscle, hypothalamus, pituitary gland, granulosa cells, abdominal fat, small intestine, and muscular stomach.

In order to analyze the potential role of GR in Yangzhou Geese laying performance, the expression levels of GR mRNA in 10 tissues (liver, ovary, heart, breast muscle, hypothalamus, pituitary gland, granulosa cells, abdominal fat, small intestine, and muscular stomach) were evaluated by qPCR. The results showed that GR was ubiquitously expressed in the 10 tested tissues (Figure 6). The highest expression levels were detected in ovary, pituitary gland, abdominal fat, and muscular stomach (Figure 6).

### 2.9. Glucocorticoid Promotes the Transcription Activity of the C But Not of the G Allele

**Figure 7:**
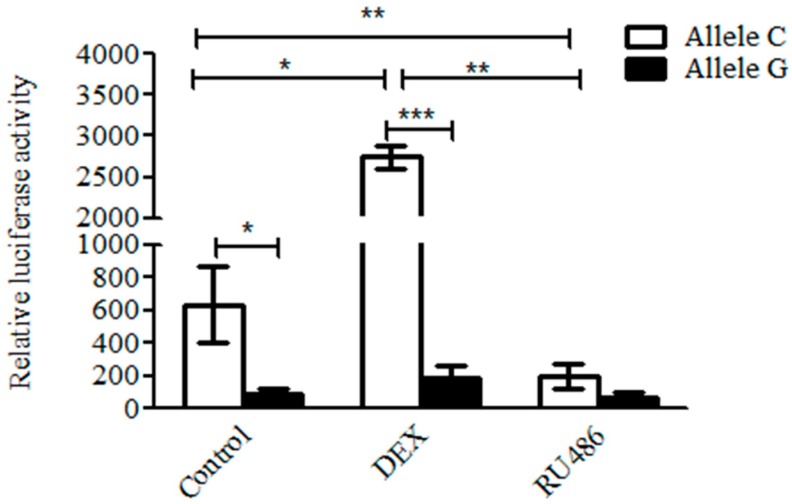
Promoters contaning different alleles of SNP c.-413C>G have respond distinctly to GR. Reporter gene assay using constructs including the C or the G alleles of SNP c.-413C>G. The reporter constructs were transfected into 293 T cell; 2.5 μM DXM was used as an agonist of GR, whereas 100 nM RU486 was used as an antagonist of GR. The data represent the mean ± SD of three independent experiments (* *p* < 0.05, ** *p* < 0.01, *** *p* < 0.001).

A reporter gene driven by the *KIAA1462* promoter with the C or G alleles was constructed to further investigate the effect of glucocorticoid and allele shift on *KIAA1462* gene expression. A 630 bp DNA fragment including c.-413C or c.-413G was subcloned into the multiple cloning sites of the PGL3 promoter vector. The relative luciferase activity of the C allele plasmid was significantly higher than that of the G allele (Figure 7) (*p* < 0.05). Furthermore, when the cells were treated with 2.5 μM dexamethasone (glucocorticoid analogue), the luciferase activity of the C allele vector was significantly increased 4.4 folds (*p* < 0.05), while no significant change for the G allele vector was observed (*p* > 0.05). When treated with 100 nM RU486, a GR antagonist, the luciferase activity of the C allele vector was remarkably reduced 3.2 folds (*p* < 0.01), whereas the G allele vector was not affected notably (*p* > 0.05). These results indicated that the c.-413C but not the c.-413G allele was highly activated promoting the transcription of the *KIAA1462* gene in response to glucocorticoid.

## 3. Discussion

The laying performance is an important economical trait in goose production. As for other poultry production, it is difficult to improve such a low-heritability trait by using direct phenotypic selection. Therefore, molecular markers or functional genes involved in the egg-laying process are vital for improving the efficiency of the laying performance. Our previous study identified a laying-related SNP in an intron of the goose *KIAA1462* gene [4]. The genetic variants of the *KIAA1462* gene are thought to be involved in human reproduction [6,7]. Nevertheless, there is no report about the potential contribution of *KIAA1462* gene to goose laying performance. In this study, we investigated whether *KIAA1462* gene and its genetic mutations affect goose reproduction and discovered a mutation involved in this process.

We firstly obtained the Yangzhou geese *KIAA1462* cDNA sequence by PCR sequencing. A 4029 bp cDNA sequence encoding a protein of 1342 amino acids consistent with the Chinese white goose corresponding protein (XM_013191711.1) was obtained from goose ovary tissue, and was found to share a high identity with the corresponding proteins of the Mallard duck (XM_005027879.2) and the chicken (XM_418578.5), i.e., 88.73% and 87.67%, respectively. According to the results showed in Figure 1 and Figure 6, *KIAA1642* is expressed in many tissues, but its interaction proteins and active pathways in granulosa cells are quite different from those in the other tissues. Thus, the relative high expression level in granulosa cells allows *KIAA1642* to execute its function effectively and specifically.

It has been confirmed that *KIAA1462* is mainly colocalized with ZO-1, a tight junction-associated plaque protein and an excellent marker for epithelial cell–cell junctions in immunofluorescence microscopy [4]. Tight junctions (TJ) not only participate in regulating the transport of essential materials and the maintenance of epithelial cell polarity [16], but also play an important role in cell proliferation and differentiation, tumor cell metastasis, and gene transcription [17]. *KIAA1462* was identified as a novel cell–cell junction-associated protein [3], which implies that *KIAA1462* may play an important role in cell–cell junctions. Cells transmit metabolite and second messengers, such as cAMP and Ca^2+^, through cell–cell junctions, which can participate in asymmetric cell divisions and affect cell proliferation and apoptosis [18]. In this study, we demonstrated that geese with the AA genotype exhibited higher egg production performance and higher transcription levels of *KIAA142* than geese with the TT genotype. The egg laying-related SNP was located in the *KIAA1642* gene, indicating the potential role of *KIAA1642* in laying performance. In order to confirm its function and study the underlying mechanism, we performed gain- and loss-of-function experiments and found that *KIAA1462* could negative regulate the expression of caspase-3 gene in vitro, which is a key factor contributing to granulosa cells apoptosis [12,19,20]. Proliferation and apoptosis of granulosa cells have been proved to be involved in follicle development and oviposition during the egg-laying period [13,14,21,22]. In this study, the higher *KIAA1462* mRNA level in ovarian and F1 follicles compared with other tissues also allows its functional role in follicular development. Therefore, the differences in laying performance among the three genotypic Yangzhou geese could be caused by the differential granulosa cell apoptosis resulting from *KIAA1462* polymorphism.

Moreover, the influence of SNP in regulating *KIAA1642* gene expression level was estimated. Because of the fact that SNP rs1714766362 is located in an intron of the *KIAA1462* gene, which means that it seldom participates in translation and contributes to the structure of the encoded protein, it was necessary to perform more experiments to explain its function in *KIAA1462* gene. Therefore, we postulated that there might be another functional mutation tightly linked with SNP rs1714766362, which could modulate the gene transcription. By comparing the promoter regions of *KIAA1462* gene between the AA and the TT individuals, we found a novel mutation, namely, c.-413C>G, in the transcription regulatory region, which was completely linked with SNP rs1714766362. The promoter with the C allele had higher transcription activity than that with the G allele. Online prediction and a subsequent EMSA experiment showed that the transcription factor GR could bind to the C allele but not to the G allele. This was further confirmed by the observation that glucocorticoid significantly activated, while RU486 suppressed, the transcription activity of a C allele vector but had no effect on a G allele vector.

GR is a glucocorticoid receptor which mediates the biological effect of glucocorticoids. Previous studies have provided insights into its role in the anti-inflammatory response, immune suppression, and metabolic regulation [23,24,25]. The GC/GR/GRE complex interacts with other nucleoproteins to mediate the transcription of target genes [26,27]. To our knowledge, this is the first report demonstrating that *KIAA1462* transcription is directly influenced by GC/GR signal. Moreover, it is interesting that this GC/GR signal can be affected by the c.-413C>G mutation in the promoter region, resulting in differential *KIAA1462* expression levels. Studies showed that hens fed corticosterone or infused with corticosterone had ovary regression and reduced weight gain and egg production [8,9]. In addition, glucocorticoids downregulated the expression of appetite-related genes in the hypothalamus of HFD-fed chicks by an AMPK–neuropeptide Y signaling pathway and a TOR pathway [10,11].

In conclusion, we identified a novel functional SNP affecting goose *KIAA1462* gene expression. The modulated *KIAA1462* expression influenced apoptosis of granulosa cells and therefore affected eggs yield. Our findings identify a novel candidate gene and a molecular marker that can be useful to remarkably improve goose laying performance.

## 4. Materials and Methods

### 4.1. Ethics Statement

Animal experiments were reviewed and approved by Nanjing Agricultural University Animal Care and Use Committee and performed in accordance with the Regulations for the Administration of Affairs Concerning Experimental Animals (China, Decree No. 2 of the State Science and Technology Commission, 14 November 1988). All efforts were made to minimize any discomfort during goose slaughtering process.

### 4.2. Animals and Samples Preparation

Two hundred and fifty-six white Yangzhou geese, provided by the breeding farm of Jiangsu Lihua Animal Husbandry Co., Ltd. (Changzhou, Jiangsu, China), were used in this study. During the experiments, geese were fed ad libitum with rice grain supplemented with green grass or water plants whenever possible. The feed was offered during daytime when the geese were released to an open area outside the house. The geese were exposed to natural lighting and temperature throughout this study. Individual laying records, during the entire egg-laying period (34 weeks), were obtained from Jiangsu Lihua Animal Husbandry Co., Ltd. Blood samples of all 256 geese were collected for DNA extraction. Sixteen laying geese with AA or TA genotypes (*n* = 8 for each genotype) were slaughtered in the peak laying period (100 days after geese began to lay eggs) to collect the tissues, including kidney, ovary, small intestine, liver, abdominal fat, muscular stomach, breast muscle, hypothalamus, pituitary gland, and heart. The tissues were immediately frozen and stored in liquid nitrogen until total RNA was extracted. The follicles of different developmental stages (F1–F5, small yellow follicle, large white follicle, small white follicle) from each ovary and granulosa cells from hierarchical follicles were separated as previously described [28].

### 4.3. Genotyping and Association Analysis

SNP rs1714766362 was genotyped for 256 Yangzhou geese by Allele-Specific PCR (AS-PCR). Two allele-specific primers and a universal primer were designed according to a protocol described previously [29,30] (Table 3). The PCR reactions were performed in 20 μL, including 10 μL r-taq, 1 μL forward primer, 1 μL reverse primer, 1 μL DNA template, and 7 μL dd H_2_O. The PCR reaction procedure was as follows: 94 °C for 5 min, 32 cycles of amplification (94 °C for 30 s, 58 °C for 30 s, and 72 °C for 15 s), and a final extension at 72 °C for 7 min. The 67 bp PCR products were subjected to electrophoresis using 3% agarose gel, and the target bands were excised under UV light. Ten PCR products were extracted and sequenced (GENEWIZ, Suzhou, China) to confirm their identity.

### 4.4. RNA Isolation and First-Strand cDNA Synthesis

Total RNA was isolated from different tissues using a Trizol reagent (Invitrogen, Carlsbad, CA, USA), according to the standard protocol, and subsequently treated with DNase I (Invitrogen). The quality of the RNA samples was evaluated by electrophoresis on 1% agarose gels. ProtoScript^®^ First Strand cDNA Synthesis kit (NEB, Beijing, China) was used to synthesize cDNA. The reverse transcription system contained 1 μg of total RNA, 2 μL of d(T)_23_ VN (50 μM), 10 μL of M-Mulv reaction mix, 2 μL of M-Mulv enzyme mix, and nuclease-free water to 20 μL. Firstly, total RNA was mixed with d(T)_23_ VN primer and nuclease-free H_2_O and then was denatured for 5 min at 70 °C. This mixture was spun briefly and taken to 0 °C. The M-Mulv reaction mix and M-Mulv enzyme mix were added to the above mixture and incubated at 42 °C for 1 h; subsequently enzyme inactivation was performed at 80 °C for 5 min. After that, we diluted the reaction products to 100 μL with 80 μL H_2_O for PCR reaction and stored the samples at −20 °C.

### 4.5. Cloning of KIAA1462 Gene Coding Sequence in Yangzhou Geese

In order to clone the full-length coding region of Yangzhou geese *KIAA1462* gene, we designed three pairs of primers (Table 3, P1, P2, P3) based on the most conserved regions of the orthologous sequence in *Anas platyrhynchos* (XM_005027879.2). The three output sequences were overlapped to obtain the contig for *KIAA1462* coding region. PCR amplification was conducted in a final volume of 50 μL, containing 2 μL first-strand cDNA, 2.5 μL each primer (10 nM), 10 μL 5× reaction buffer, 0.5 μL High-Fidelity DNA Polymerase (NEB, Beijing, China), 1 μL10 mM dNTP, and 31.5 μL nuclease-free water. The PCR reactions were performed as follows: 98 °C for 30 s, 32 cycles of amplification (98 °C for 10 s, 56 °C for 30 s and 72 °C for 2 min), and a final extension at 72 °C for 2 min. The PCR products were subjected to electrophoresis on 2% agarose gel, and the target bands were excised under UV light and purified using the E.Z.N.A. Gel Extraction Kit (Omega Bio-Tek, Doraville, GA, USA), as recommended by the supplier. The purified products were cloned into the Peasy-T3 vector (TransGen Biotech, Beijing, China) and then transfected into the Trans-T1 phage-resistant chemically competent cells (TransGen Biotech, Beijing, China). PCR was used to identify the positive clones. The clones of different cDNA fragments were sequenced by a company (GENEWIZ, Suzhou, China).

### 4.6. Construction of pEGFP-KIAA1462-N1 Expression Vector

The primer pair P4 that include EcoR I and Sma I restriction sites (underlined residues) (Table 3) was used to amplify the full-length coding sequence of *KIAA1462* gene. The inserts were released by restriction enzyme digestion and covalently linked in-frame at the corresponding restriction sites in the multiple cloning site (MCS) of the commercial pEGFP-N1, encoding a red-shifted variant of wild-type GFP (BD Biosciences Clontech, Franklin Lakes, NJ, USA). All constructs were verified by sequencing to ensure in-frame integrity at the pEGFP-*KIAA1462* junction. The ligation-Free Cloning Kit (ABM, Richmond, BC, Canada) was used to complete the ligation between the target DNA fragment and the linearized vector, following the manufacturer’s instruction.

### 4.7. Quantitative Real-Time PCR Analysis

Quantitative real-time PCR (qPCR) was used to determine the expression of *KIAA1462* (amplified with primer P5, Table 3) in various tissues of geese including kidney, ovary, small intestine, liver, abdominal fat, muscular stomach, breast muscle, heart, hypothalamus, pituitary gland, as well as granulosa cells from different developmental stages of follicles (F1–F5, small yellow follicle, large white follicle, small white follicle). The expression of *KIAA1462* and caspase-3 gene in granulosa cells were also detected by qPCR in overexpression or knockdown experiments. SYBR^®^ Green Master Mix (Vazyme, Nanjing, China) was used in a StepOne Plus Real-Time PCR system (Applied Biosystems, Foster City, CA, USA). The PCR reaction (20 μL) consisted of 1 μL cDNA, 0.4 μL of each primer (10 μmol), 0.4 μL ROX Reference Dye, 10 μL SYBR Green Master Mix, and 7.8 μL nuclease-free water. Amplification conditions were as followed: pre-denaturation at 95 °C for 5 min, 40 cycles of amplification (95 °C for 10 s and 60 °C for 30 s). A melt curve analysis was performed from 60 °C to 95 °C by reading plate every 0.1 °C. Each sample was analyzed three times. Gene expression levels were calculated by the 2^−ΔΔ*C*t^ method using GAPDH as an internal control [31,32,33,34].

### 4.8. Granulosa Cell Culture and KIAA1462 Gene Overexpression

The in vitro experiments were conducted according to a protocol described previously [2]. Briefly, the harvested granulosa sheets from pre-ovulatory follicles (F5–F1) were dispersed with type II collagenase (Sigma-Aldrich Co., LLC, St. Louis, MO, USA) at 37 °C for 10–15 min. Then, the cells were washed with Hanks’ balanced salt solution (Gibco, Life Technologies, Carlsbad, CA, USA) and filtered by a 75 μm cell strainer.

The filtered liquor was centrifuged at 924× *g* for 5 min. After that, the granulosa cells were resuspended in Dulbecco’s modified Eagle’s medium/nutrient mixture (DMEM/F12) containing 3% fetal bovine serum (ScienCell Research Laboratories, Carlsbad, CA, USA). The freshly isolated granulosa cells were diluted with media to a concentration of 5 × 10^5^ cells/mL, cell viability was assessed by the trypan blue dye exclusion test (Invitrogen, Life Technologies, Carlsbad, CA, USA), and the cells were incubated at 37 °C in 5% CO_2_ in a humidified incubator. After 24 h, the granulosa cells were seed in 12-well plates. When grown to 60–70% confluency, the cells were transfected by 5 μL Lipofectamine 2000 (Invitrogen Life Technologies Inc., Carlsbad, CA, USA) with 2 μg pEGFP-*KIAA1462*-N1. After transfection, the cells were kept in the incubator for additional 24 h and then were lysed with 500 μL Trizol (Invitrogen, Carlsbad, CA, USA) for total RNA extraction.

### 4.9. siRNA Preparation and Transfection

Three small interfering RNAs were designed with the common sequence of *KIAA1462* CDS (Coding Sequence) (Table 3, siRNA1847, siRNA1200, siRNA2180). The siRNAs were synthesized by Shanghai GenePharma Co., Ltd. (Shanghai, China). When granulosa cells reached 60–70% confluency in 12-well plates, 200 pmol siRNA was transfected by 5 μL Lipofectamine 2000, following the manufacturer’s instructions. After transfection, the cells were kept in the incubator for additional 24 h and then were lysed with 500 μL Trizol for total RNA extraction.

### 4.10. Mutation Detection in KIAA1462 Promoter Region

Direct sequencing of the 3 kb promoter region of *KIAA1462* gene was conducted for individuals with the AA genotype and the TT genotype for SNP rs1714766362. The sequencing results were compared by DNAMAN 8.0 (Lynnon Corporation, Montreal, QC, Canada).

### 4.11. Electrophoretic Mobility Shift Assay (EMSA)

Goose granulosa cells were grown in 15 cm culture plates until they reached 95% confluency. The plates were then sealed with parafilm and immersed in a water bath at 42.5 °C for 1.5 h. Nuclear extracts from these cells were prepared according to a standard protocol [35]. The sequences of the three probes are listed in Table S1. EMSA experiments were carried out using the Chemiluminescent EMSA Kit (Beyotime Biotechnology, Shanghai, China), as recommended by the supplier. In brief, 2 μL EMSA/Gel-shift binding buffer was mixed with 5 μg nuclear extract at room temperature for 10 min, then 1 μL biotin-labeled probe was added, and hybridization was carried out for 20 min at ambient temperature. The mixtures were then loaded into a 6.5% Polyacrylamide gel, separated by electrophoresis at 4°C, and transferred onto a nylon membrane [36]. As competitors, non-labeled oligo nucleotides were incubated with nuclear extracts before adding the labeled probe. All EMSA experiments were repeated twice for confirmation of the results.

### 4.12. Dual Luciferase Reporter Assay

The 630 bp fragments including the C or G alleles of SNP (c.-413C>G) were cloned into PGL3-promoter vector (Promega, Madison, WI, USA). Two plasmids were co-transfected with Dexamethasone (2.5 μM) [37,38] and RU486 (Inhibitor of GR, 100 nM) [39,40] into 293 T cells by Lipofectamine 2000 (Invitrogen Life Technologies Inc., Carlsbad, CA, USA). The cells were kept in the incubator for 24 h, and the relative luciferase activity was measured by Dual Luciferase Assay System [41,42].

### 4.13. Bioinformatics Analysis

Sequence chromatograms were examined and edited by Chromas Version 2.23 (http://technelysium.com.au/). The sequence comparisons were conducted by DNAMAN 8.0 (http://www.lynnon.com/). Related sequences were identified with ensembl (http://www.ensembl.org/index.html), Genbank (http://www.ncbi.nlm.nih.gov/genbank), and BLAST (http://www.ncbi.nlm.nih.gov/BLAST/). The identification of ORFs (Open Reading Frame) was performed by using the ORF Finder tool of NCBI (http://www.ncbi.nlm.nih.gov/gorf/gorf.html). Protein Sequences were translated using DNAStar 5.02 (DNASTAR Inc.). Phylogenetic tree construction was performed by MEGA 5.0 (http://www.megasoftware.net/). The molecular weight and isoelectric point of the protein were analyzed using the ExPASy ProtParam tool (http://www.expasy.org/tools/protparam.html). The transcription factor was predicted by using the transcription factor prediction tool MatInspector (http://www.genomatix.de/products/MatInspector/index.html).

## Figures and Tables

**Table 1:** The association analysis between different genotypes of SNP rs1714766362 and total number of eggs within a 34-week egg-laying period in Yangzhou geese.

Genotype	Number of Geese	Number of Eggs	Lower Mean	Upper Mean	*p* Value
AA	170	79.60 ± 11.08 ^a^	77.94	81.47	0.04
TA	73	77.61 ± 11.11 ^a^^,b^	74.79	80.28	
TT	13	73.00 ± 9.61 ^b^	69.62	76.23	
Total	256	78.73 ± 11.61	77.43	80.11	

The egg number is presented as mean ± SD. Multiple comparisons were performed using the Duncan multiple-range test; ^a,b^ means with different superscripts in the same column are significantly different (*p* < 0.05).

**Table 2:** Homology analysis of goose *KIAA1462* gene coding sequence (CDS) compared to other species.

Species	Nucleotide Accession Number	Amino Acid Accession Number	Nucleotide	Amino Acid
*Platyrhynchos* (Mallard)	XM_005027879.2	XP_005027936	88.73%	86.91%
*Gallus gallus* (chicken)	XM_418578.5	XP_418578.4	87.67%	85.80%
*Meleagris gallopavo* (turkey)	XM_003206999.2	XP_003207047.2	86.68%	84.01%
*Columba livia* (Rock pigeon)	XM_005504208.1	XP_005504265.1	83.74%	80.19%
*Canis lupus familiaris* (Dog)	XM_535151.5	XP_535151.2	52.60%	40.56%
*Homo sapiens* (Human)	NM_020848.2	NP_065899.1	55.01%	40.68%
*Ovis aries* (Sheep)	XM_004014272.3	XP_004014321.1	51.04%	35.64%
*Bos taurus* (Cattle)	NM_001082474.1	NP_001075943.1	50.68%	36.66%
*Sus scrofa* (Pig)	XM_003130706.5	XP_003130754.3	52.06%	36.67%

Information of *KIAA1462* genes and proteins was downloaded from Genbank. Homology analysis was performed by using the BLAST program on this web.

**Table 3:** Primers used in this study.

Name	Sequences (5′→3′)	Function	Size (bp)	Tm (°C)
S1	GCTGACAGCTCATTTGATA	AS-PCR	67	58
S2	GCTGACAGCTCATTTGATT	AS-PCR		
AS	CAGGATCACGTCCTCAAC	AS-PCR		
P1-F	ATGTTCAGTGTCGAGGACCTCC	Partial cDNA sequences	1305	58
P1-R	AACAGAACGCAGGTAGTCA			
P2-F	GACCGCCTGCGAATAGTGT	Partial cDNA sequences	1424	56
P2-R	CGTTTCCAACCTCCCACC			
P3-F	CTGAAGCCCGTAAGTCG	Partial cDNA sequences	1300	56
P3-R	CTATTTGAGCGTCATTACGTGGG			
P4-F	CGAATTCCATGTTCAGTGTCGAGGACCTCC	KIAA1462 expression vector	4029	62
P4-R	CCCCGGGGCTATTTGAGCGTCATTACGTGGG			
P5-F	AGCATGAGGTGCGTGGAGATG	q-PCR	200	60
P5-R	CTCCAAACCCGAGTCTTGAACG			
caspase-3-F	CTGGTATTGAGGCAGACAGTGG	q-PCR	158	50
caspase-3-R	CAGCACCCTACACAGAGACTGAA			
GAPDH-F	GCTGATGCTCCCATGTTCGTGAT	q-PCR	86	60
GAPDH-R	GTGGTGCAAGAGGCATTGCTGAC			
GR-F	CTCTGGGTGTCATTACGGTGTT	q-PCR	230	62
GR-R	CATTAGCTTGCTGGATTCCTTT			
KIAA1462-siRNA-1200	GCCUGCGAAUAGUGUUGAATT	KIAA1462 knockdown experiments		
	UUCAACACUAUUCGCAGGCTT			
KIAA1462-siRNA-1847	CCCAGAGCCCUGAUAAGAATT	KIAA1462 knockdown experiments		
	UUCUUAUCAGGGCUCUGGGTT			
KIAA1462-siRNA-2180	CCAAACUGCUGUCUCCAAATT	KIAA1462 knockdown experiments		
	UUUGGAGACAGCAGUUUGGTT			
P6-F	TGGTTGATGTCCTGCGGG	Partial promoter sequences	2440	59
P6-R	CCACCCTTACAATAAAGCACA			
P7-S1	TCCCAGAATACAGAGCACTCC	AS-PCR	265	58
P7-S2	TCCCAGAATACAGAGCACTCG	AS-PCR		
P7-AS	CCACCCTTACAATAAAGCACATC	AS-PCR		
P8-F	GGGGTACCCCGGGAATCATTGAGACACGACA	Dual luciferase reporter vector	630	64
P8-R	CCCTCGAGGGGCTGCTGAAGTGAAGGGTTT			

F: Forward primers; R: Reverse primers.

## Supplementary Materials

Supplementary materials can be found at http://www.mdpi.com/1422-0067/19/5/1531/s1.

## Abbreviations

AMPK	Adenosine 5′-monophosphate (AMP)-activated protein kinase
AS-PCR	Allele-Specific PCR
CDS	Coding Sequence
DMEM	Dulbecco’s modified Eagle’s medium
EMSA	Electrophoretic Mobility Shift Assay
GR	Glucocorticoid Receptor
HFD	High-Fat Diet
JCAD	Junctional Protein Associated with Coronary Artery Disease
ORF	Open Reading Frame
Q-PCR	Quantitative Real Time Polymerase Chain Reaction
RAD sequencing	Restriction Site-Associated Sequencing
SNP	Single-Nucleotide Polymorphism
TOR	Target of Rapamycin

## References

[1] Qin Q., Sun A., Guo R., Lei M., Ying S., Shi Z. (2013). The characteristics of oviposition and hormonal and gene regulation of ovarian follicle development in Magang geese. Reprod. Biol. Endocrinol..

[2] Hu S., Liu H., Pan Z., Xia L., Dong X., Li L., Xu F., He H., Wang J. (2014). Molecular cloning, expression profile and transcriptional modulation of two splice variants of very low density lipoprotein receptor during ovarian follicle development in geese (Anser cygnoide). Anim. Reprod. Sci..

[3] Akashi M., Higashi T., Masuda S., Komori T., Furuse M. (2011). A coronary artery disease-associated gene product, JCAD/KIAA1462, is a novel component of endothelial cell-cell junctions. Biochem. Biophys. Res. Commun..

[4] Yu S., Chu W., Zhang L., Han H., Zhao R., Wu W., Zhu J., Dodson M.V., Wei W., Liu H. (2015). Identification of laying-related SNP markers in geese using RAD sequencing. PLoS ONE.

[5] Murdock D.G., Bradford Y., Schnetz-Boutaud N., Mayo P., Allen M.J., D’Aoust L.N., Liang X., Mitchell S.L., Zuchner S., Small G.W., Gilbert J.R. (2013). KIAA1462, a coronary artery disease associated gene, is a candidate gene for late onset Alzheimer disease in APOE carriers. PLoS ONE.

[6] Chowdhury R., Bois P.R., Feingold E., Sherman S.L., Cheung V.G. (2009). Genetic analysis of variation in human meiotic recombination. PLoS Genet..

[7] Boyd J., Luo B., Peri S., Wirchansky B., Hughes L., Forsythe C., Wu H. (2013). Whole exome sequence analysis of serous borderline tumors of the ovary. Gynecol. Oncol..

[8] El-lethey H., Jungi T.W., Huber-Eicher B. (2001). Effects of feeding corticosterone and housing conditions on feather pecking in laying hens (*Gallus gallus* domesticus). Physiol. Behav..

[9] Williams J.B., Etches R.J., Rzasa J. (1985). Induction of a pause in laying by corticosterone infusion or dietary alterations: Effects on the reproductive system, food consumption and body weight. Br. Poult. Sci..

[10] Liu L., Wang X., Jiao H., Zhao J., Lin H. (2015). Glucocorticoids inhibited hypothalamic target of rapamycin in high fat diet-fed chicks. Poult. Sci..

[11] Liu L., Song Z., Jiao H., Lin H. (2014). Glucocorticoids increase NPY gene expression via hypothalamic AMPK signaling in broiler chicks. Endocrinology.

[12] Choudhary G.S., Al-Harbi S., Almasan A. (2015). Caspase-3 activation is a critical determinant of genotoxic stress-induced apoptosis. Methods Mol. Biol..

[13] Hrabia A., Leśniak-Walentyn A., Sechman A., Gertler A. (2014). Chicken oviduct-the target tissue for growth hormone action: Effect on cell proliferation and apoptosis and on the gene expression of some oviduct-specific proteins. Cell Tissue Res..

[14] Chen F., Jiang Z., Jiang S., Li L., Lin X., Gou Z., Fan Q. (2016). Dietary vitamin A supplementation improved reproductive performance by regulating ovarian expression of hormone receptors, caspase-3 and Fas in broiler breeders. Poult. Sci..

[15] Werner T. (2000). Computer-assisted analysis of transcription control regions. Matinspector and other programs. Methods Mol. Biol..

[16] Balkovetz D.F. (2007). Opening Pandora’s box in the tight junction. J. Am. Soc. Nephrol..

[17] Georgiadis A., Tschernutter M., Bainbridge J.W., Balaggan K.S., Mowat F., West E.L., Munro P.M., Thrasher A.J., Matter K., Balda M.S. (2010). The tight junction associated signalling proteins ZO-1 and ZONAB regulate retinal pigment epithelium homeostasis in mice. PLoS ONE.

[18] Flores I., Jones D.R., Merida I. (2000). Changes in the balance between mitogenic and antimitogenic lipid second messengers during proliferation, cell arrest, and apoptosis in T-lymphocytes. FASEB J..

[19] Suzuki A., Tsutomi Y., Akahane K., Araki T., Miura M. (1998). Resistance to Fas-mediated apoptosis: Activation of caspase 3 is regulated by cell cycle regulator p21WAF1 and IAP gene family ILP. Oncogene.

[20] Yakovlev A.G., Ota K., Wang G., Movsesyan V., Bao W.L., Yoshihara K., Faden A.I. (2001). Differential expression of apoptotic protease-activating factor-1 and caspase-3 genes and susceptibility to apoptosis during brain development and after traumatic brain injury. J. Neurosci..

[21] Johnson A.L., Woods D.C. (2009). Dynamics of avian ovarian follicle development: Cellular mechanisms of granulosa cell differentiation. Gen. Comp. Endocrinol..

[22] Johnson P.A. (2012). Follicle selection in the avian ovary. Reprod. Domest. Anim..

[23] Barnes P.J. (1998). Anti-inflammatory actions of glucocorticoids: Molecular mechanisms. Clin. Sci..

[24] Tsurufuji S., Sugio K., Takemasa F. (1979). The role of glucocorticoid receptor and gene expression in the anti-inflammatory action of dexamethasone. Nature.

[25] Turner J.D., Schote A.B., Macedo J.A., Pelascini L.P., Muller C.P. (2006). Tissue specific glucocorticoid receptor expression, a role for alternative first exon usage?. Biochem. Pharmacol..

[26] Itani O.A., Liu K.Z., Cornish K.L., Campbell J.R., Thomas C.P. (2002). Glucocorticoids stimulate human sgk1 gene expression by activation of a GRE in its 5′-flanking region. Am. J. Physiol. Endocrinol. Metab..

[27] McKay L.I., Cidlowski J.A. (1999). Molecular control of immune/inflammatory responses: Interactions between nuclear factor-kappa B and steroid receptor-signaling pathways. Endocr. Rev..

[28] Gilbert A.B., Evans A.J., Perry M.M., Davidson M.H. (1977). A method for separating the granulosa cells, the basal lamina and the theca of the preovulatory ovarian follicle of the domestic fowl (*Gallus domesticus*). J. Reprod. Fertil..

[29] Bustos A.D., Rubio P., Jouve N. (2000). Molecular characterisation of the inactive allele of the gene Glu-A1 and the development of a set of AS-PCR markers for HMW glutenins of wheat. Theor. Appl. Genet..

[30] Pauciullo A., Gallo D., Colimoro L. (2008). Genotyping at the CSN1S1 locus by PCR-RFLP and AS-PCR in a Neapolitan goat population—Small Ruminant Research. Small Rumin. Res..

[31] Alsiddig M.A., Yu S.G., Pan Z.X., Widaa H., Badri T.M., Chen J., Liu H.L. (2017). Association of single nucleotide polymorphism in melatonin receptor 1A gene with egg production traits in Yangzhou geese. Anim. Genet..

[32] Kang B., Guo J.R., Yang H.M., Zhou R.J., Liu J.X., Li S.Z., Dong C.Y. (2009). Differential expression profiling of ovarian genes in prelaying and laying geese. Poult. Sci..

[33] Nascimento C.S., Barbosa L.T., Brito C., Fernandes R.P., Mann R.S., Pinto A.P., Oliveira H.C., Dodson M.V., Guimaraes S.E., Duarte M.S. (2015). Identification of suitable reference genes for real time quantitative polymerase chain reaction assays on pectoralis major muscle in chicken (*Gallus gallus*). PLoS ONE.

[34] Schybli M., Sigrist B., Hess M., van Leerdam B., Hoop R.K., Vogtlin A. (2014). Development of a new real-time polymerase chain reaction assay to detect Duck adenovirus A DNA and application to samples from Swiss poultry flocks. J. Vet. Diagn. Investig..

[35] Andrews N.C., Faller D.V. (1991). A rapid micropreparation technique for extraction of DNA-binding proteins from limiting numbers of mammalian cells. Nucleic Acids Res..

[36] Lo P.H., Urabe Y., Kumar V., Tanikawa C., Koike K., Kato N., Miki D., Chayama K., Kubo M., Nakamura Y. (2013). Identification of a functional variant in the MICA promoter which regulates MICA expression and increases HCV-related hepatocellular carcinoma risk. PLoS ONE.

[37] Attarzadeh-Yazdi G., Shipston M.J., Antoni F.A. (2008). Dex-ras1 and serum- and glucocorticoid-inducible protein kinase 1: Regulation of expression by dexamethasone in HEK293 cells. Neurochem. Res..

[38] Guney S., Schuler A., Ott A., Hoschele S., Zugel S., Baloglu E., Bartsch P., Mairbaurl H. (2007). Dexamethasone prevents transport inhibition by hypoxia in rat lung and alveolar epithelial cells by stimulating activity and expression of Na^+^-K^+^-ATPase and epithelial Na^+^ channels. Am. J. Physiol. Lung Cell. Mol. Physiol..

[39] Hori T., Jin L., Fujii A., Furihata T., Nagahara Y., Chiba K., Hosokawa M. (2012). Dexamethasone-mediated transcriptional regulation of rat carboxylesterase 2 gene. Xenobiotica.

[40] Stevens A., Garside H., Ray D. (2002). RU486, the glucocorticoid receptor (GR) antagonist, recruits NCoR, but not SRC-1: Explaining type II antagonism. Endocr. Abstr..

[41] Harger J.W., Dinman J.D. (2003). An in vivo dual-luciferase assay system for studying translational recoding in the yeast Saccharomyces cerevisiae. RNA.

[42] Noguchi T., Ikeda M., Ohmiya Y., Nakajima Y. (2012). A dual-color luciferase assay system reveals circadian resetting of cultured fibroblasts by co-cultured adrenal glands. PLoS ONE.

