# Supplementary material for: A Functional Mutation in KIAA1462 Promoter Decreases Glucocorticoid Receptor Affinity and Affects Egg-Laying Performance in Yangzhou Geese
Source: Int J Mol Sci. 2018 May 21;19(5):1531. doi: 10.3390/ijms19051531 (PMC5983849; doi:10.3390/ijms19051531)
Supplement: Supplementary file 1 [file ijms-19-01531-s001.pdf]

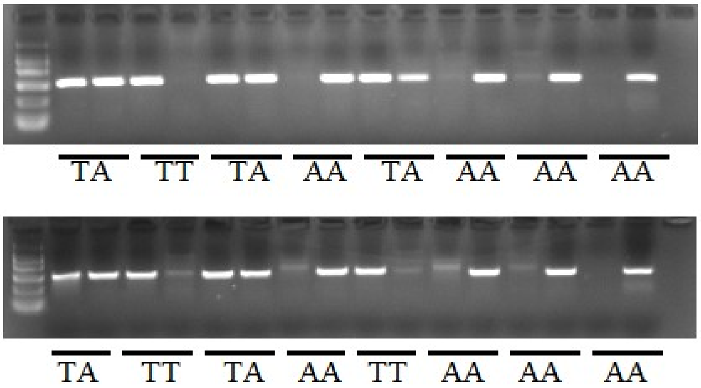

Figure S1. Genotyping of rs1714766362 SNP in *KIAA1462* gene by AS-PCR.

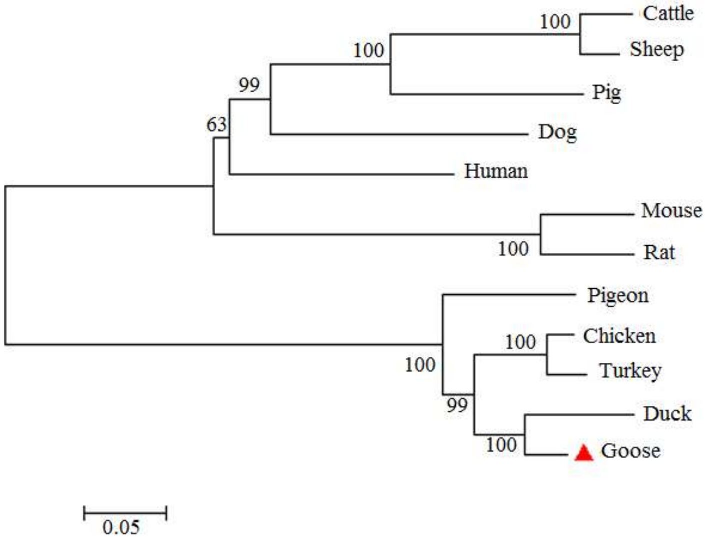

Figure S2. The phylogenetic tree of *KIAA1462* protein was constructed with the neighbour-.joining method by using MEGA5. A 1000 bootstrap replication was chosen to test the reliability of each branch. Bootstrap values are indicated as numbers at the branch nodes. Aminoacid sequences of *KIAA1462* for these species were downloaded from protein database of NCBI. Their corresponding accession numbers are same as given in Table 4.

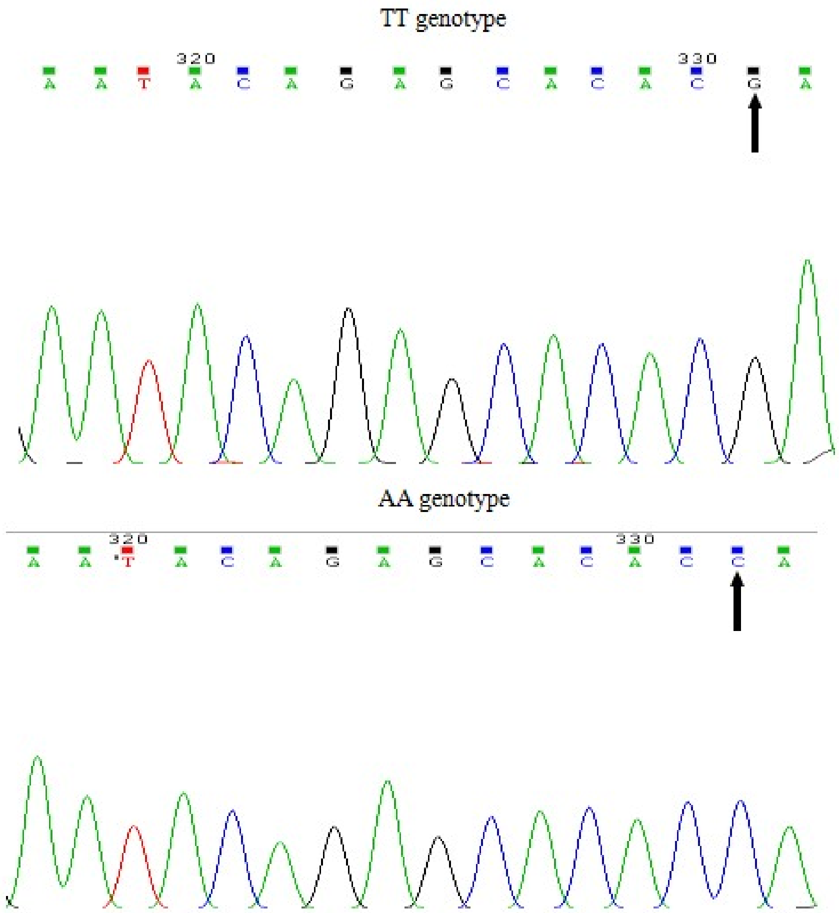

Figure S3. Direct DNA sequencing of the 3-kb promoter region using DNA from AA genotype individuals and TT genotype individuals of SNP rs1714766362. A mutation (c.-413C>G) 413-bp upstream of the start codon point of *KIAA1462* gene was completely linked with SNP rs1714766362: it was C in AA genetype individuals and G in TT genetype individuals.

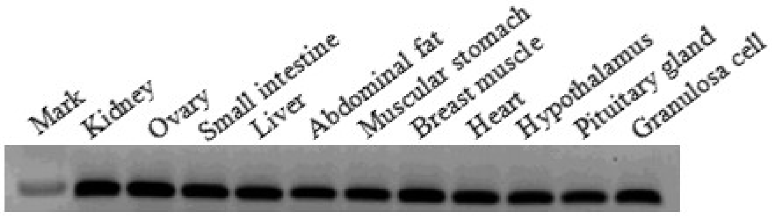

Figure S4. mRNA expression levels of goose GAPDH gene in different tissues was detected by Semi-quantitative RT-PCR.

Table S1. The sequences of each oligo used in the EMSA.

		Forward	Reverse
EMSA	**SNP(g.413C>G)-C**	**AAACAGCTTTCCTCTGGTGTGCTCTGTATTC**	**GAATACAGAGCACACCAGAGGAAAGCTGTTT**
	**SNP(g.413C>G)-G**	**AAACAGCTTTCCTCTCGTGTGCTCTGTATTC**	**GAATACAGAGCACACGAGAGGAAAGCTGTTT**
	**GR（GRE）**	**TCGACTGTACAGGATGTTGCTACT**	**AGCTGACATGTCCTACAACGATGA**
